# Removal of Foreign Bodies from the Ear

**Published:** 1876-05

**Authors:** 


Removal of Foreign Bodies from the Ear.—Mr. W. Rivington says, in the *British Medical Journal:* From the time of my first connection with the Aural Department at the London Hospital I have used no other means of extraction of foreign bodies than the syringe, aided occasionally by chloroform, the dependent position of the organ, and the use of a small pair of curved forceps as soon as the substances appeared near the external end of the meatus; and I have never failed in procuring their ejection. Various kinds of foreign bodies, including peas, beans, pebbles, glass-beads, pins, etc., have been removed in this way, and on several occasions after previous efforts by the same method or other methods had been unrewarded by success. It is the custom, I know, to make use of special forms of extractors, and instrument-makers vend a rude implement with a bent steel eye, which finds its way into cases of instruments fitted up for the receiving-rooms at hospitals. From the incautious use of such a weapon, I have seen irreparable damage done to the membrana tympani, combined with displacement of the malleus and incus, and I cannot but think that it should be banished from the surgical armamentarium.

To sum up, the procedure in cases of foreign body in the ear should be as follows:

1. Examine the ear carefully by direct light and with a speculum and mirror, to determine the presence, position, size, nature and peculiarities of the substance.

2. If the patient be a child, and refractory or timid, place him on a couch, give ether or chloroform, and use the syringe, turning the affected ear downward. This manœuvre may be aided, as Mr. Field suggests, by drawing the auricle upward and backward, and applying the nozzle of the syrings to the upper wall of the passage.

3. If the foreign substance does not fall out, as it usually does, after a little patience, but stops near the orifice of the meatus, a fine pair of forceps may be used to withdraw it.

4. A needle or a pin, or other elongated body which does not fill the passage, may be readily taken out with forceps through the speculum, or by the aid of a direct light.—*Medical and Surgical Reporter.*

